# Prevention of Tubercles

**Published:** 1842-03

**Authors:** 


*Prevention of Tubercles.*—M. Coster has submitted several animals to various experiments, for the purpose of determining how far the formation of tubercles may be prevented by diet, but principally by the administration of medicines with their food. Some rabbits were fed in the open air, and in the usual manner; another set were shut up in narrow boxes, in a cold, moist place, and deprived of light, air, &c.; they were fed on potatoes, turnips, &c. A third set were placed in the same circumstances as the latter, and nourished in the same way, but they were given every second day some bread containing nine grains of carbonate of iron. The animals were killed after the lapse of five months. The first set were healthy; the second set had tubercles in the lungs or other parts of the body; while the third set remained completely free from any trace of tubercle. M. Coster assures us that he has performed similar experiments on dogs, chickens, &c., and invariably found that the bread containing iron prevented the formation of tubercles.—*Provincial Med. and Surg. Jour.* June 12, 1841, from *Bull. de l'Acad.* No. 13.

